# Study on Purification Technology of Silicon Carbide Crystal Growth Powder

**DOI:** 10.3390/ma15228190

**Published:** 2022-11-18

**Authors:** Guofeng Fan, Tie Li, Lili Zhao, Shengtao Zhang

**Affiliations:** 1 School of Chemistry and Chemical Engineering, Harbin Institute of Technology, Harbin 150001, China; 2 Soft-Impact China (Harbin), Ltd., Harbin 150028, China; 3 Harbin KY Semiconductor, Inc., Harbin 150028, China

**Keywords:** silicon carbide, powder purification, powder heat treatment

## Abstract

Silicon carbide (SiC) is a wide-bandgap (WBG) semiconductor material, and its preparation process has strict requirements on the purity of raw materials. A self-developed medium-frequency induction heating furnace was used to carry out powder heat treatment and purification experiments on SiC powder to improve the purity of the powder. Samples with 3.5N purity were analyzed using XRD and GDMS characterization methods. It was found that under conditions of high-temperature (2200 °C) and long-time (50 h) processing, the impurity removal effect was quite good, but the powder loss was as high as 53.42%. The powder loss during the low-temperature (less than 2050 °C) and short-time process was less than 1.5%, but the purification effect was not substantial. After a prolonged processing time, the purification effect of low-temperature heat treatment conditions was improved, but the powder loss was also increased to 30%. In contrast, segmented purification processing at a low temperature in the early stage and a high temperature in the later stage achieved a good purification effect. On the premise of maintaining the utilization rate of raw materials, a 5N-purity SiC source was successfully prepared. The test results show that the contents of free Si, free C and free oxygen impurities were reduced to less than 0.01%, and the contents of Al, B, Fe, Mg, Na, Ti and other impurities were less than 1.15 ppm, which is close to the ppb level.

## 1. Introduction

As a representative of wide-bandgap semiconductor materials, silicon carbide (SiC) has been the focus of research because of its excellent performance. Single-crystal SiC has excellent physical and chemical properties. It is an ideal substrate material for making high-power, high-temperature, high-frequency, anti-radiation devices [1]. It has broad application prospects in the power electronics, transportation, clean energy, national defense and military fields. However, the application of SiC substrate materials is limited by the difficulty of obtaining high-quality single crystals. Therefore, it is very important to increase the quality of SiC crystals. SiC powder is the raw material for preparing single-crystal SiC. Its impurity content greatly affects the quality of single crystals, including metal elements such as aluminum and iron, nonmetallic elements such as boron and other group III and V elements, whose existence will result in dislocation defect proliferation, etc. For semi-insulating SiC, impurities also directly affect its resistivity [2,3,4,5,6]. There are different technologies to prepare silicon carbide powder: for example, some researchers have used the sol–gel method to synthesize nanometer 3C silicon carbide powder [7,8]. However, the large-scale preparation process is still the Acheson method [9]. Limited by production technology and the production process, industrial SiC powder usually contains high concentrations of impurities such as Al and B. If the industrial high-purity SiC powder is used directly as the raw material for single-crystal growth, impurities in the raw material will deposit into the single crystal and form various defects. The related defects will not only damage the integrity of the crystal lattice, affecting the quality and properties of the crystal, but also directly lead to the failure of subsequent single-crystal growth [10]. Therefore, before a single crystal can grow, the SiC powder source must be purified by high-temperature treatment. On the one hand, the impurities of Al, B and free silicon in the raw material can be effectively removed. On the other hand, the powder forms a frame structure after high-temperature treatment, which prevents the instability of the material transport rate in the subsequent single-crystal growth process and affects the final long-crystal results [11,12].

Currently, the commonly used powder sintering and purification process is mainly sintering and purification around the temperature of crystal growth. Different purification modes can achieve different purification effects, and some of these methods can improve the purity of raw materials to a higher level. For example, Deng designed two modes of purification, which both increased the original 97% purity of the powder to more than 99% [13,14,15,16]. However, there are still many problems with single high- or low-temperature conditions. Under high-temperature and long-time processing conditions, the SiC powder will undergo obvious recrystallization and graphitization during the heat treatment and purification process. The impurities in the powder source volatilize significantly, but the powder loss is serious. Powder with lower-temperature heat treatment has lower mass loss but only removes the impurities on the surface of the powder source, meaning that the purification effect is poorer. In this study, 3.5N-purity SiC powder was used as the raw material, and four different heat treatment processes were designed based on the advantages of the two processes. A low-mass-loss and high-purity SiC raw material heat treatment process was obtained, and the purity reached about 5N when tested by GDMS, which could be used for high-quality single-crystal SiC growth.

## 2. Experiment

**Figure 1:**
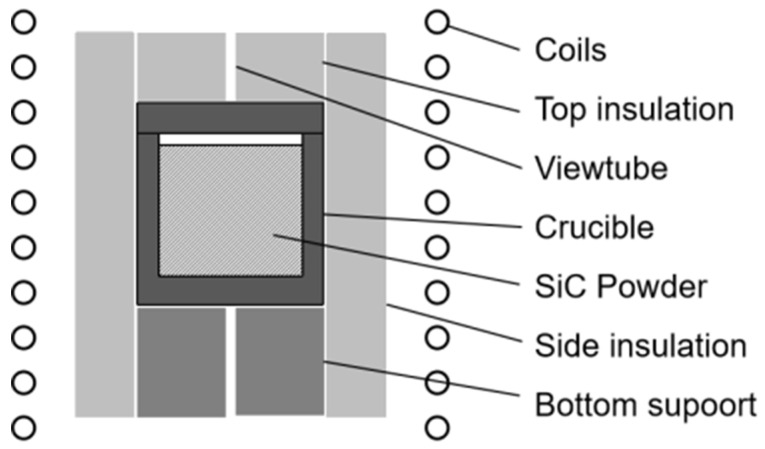
Schematic diagram of the furnace.

Induction heating has the advantage of high heating efficiency. In this experiment, a self-designed medium-frequency induction heating furnace was used, and the load was induction-heated at a frequency of 10 kHz. The basic assembly is shown in Figure 1. The assembly is composed of different high-purity graphite materials structures in a protective atmosphere, which can effectively prevent pollution by other impurities from the environment during the whole heating process. The graphite crucible or heater placed in the center of the furnace cavity is made of graphite R6510 (SGL Group, Wiesbaden, Germany), with graphite soft felt GFA5 surrounding it. The heater is supported by a graphite support structure made of graphite rigid felt MFA. SiC powder is placed inside the crucible. All of the graphite materials mentioned above are from SGL, and parameters can be found from public information. According to the principle of induction heating, the graphite heater is induced by an alternating magnetic field to generate dense eddy currents at a certain depth on the surface, which heat the SiC powder placed inside. A temperature-measuring viewport is reserved in the center above the graphite heater to measure the temperature.

**Figure 2:**
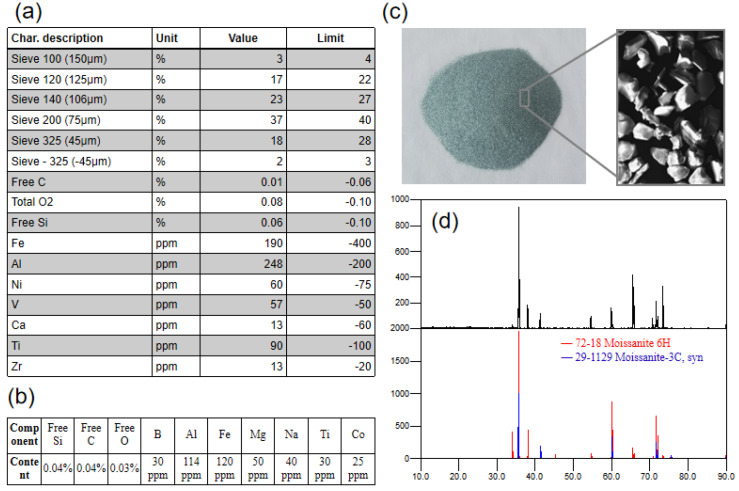
(**a**) Factory specifications; (**b**) GDMS results; (**c**) powder; (**d**) XRD patterns.

SIKA E300 SiC powder was used as the raw material in this study. The general factory specifications and test results for this type of powder are shown in Figure 2. The particle size of the powder was mostly between 75 μm and 125 μm. This batch of SiC powder looked green (Figure 2c), and XRD (X-ray diffraction, Panalytical, Almelo, Holland) detection showed that it was mainly composed of 6H and 3C polytypes (Figure 2d). The factory specifications showed that the powder particle size of this batch was mostly around 100 μm, with Al and Fe impurities reaching 248 ppm and 190 ppm, respectively (Figure 2a). More details on the impurity content were obtained by GDMS (glow discharge mass spectrometer, Nu Instruments, Wrexham, UK) detection (Figure 2b). The concentrations of Al and Fe reached 114 ppm and 120 ppm, respectively, and other elements, such as B, Mg, Na, Ti and Co, were around 30–50 ppm. The test results show that the impurity content of the powder in this batch was lower than the upper limit of the specification, which means the powder meets specification requirements. However, the contents of free Si, free C and free O, as well as Al, B and Fe, in the powder were still at high levels, which implies that there are potential problems in the direct application of the powder for the preparation of semiconductor-grade high-quality single-crystal SiC.

**Figure 3:**
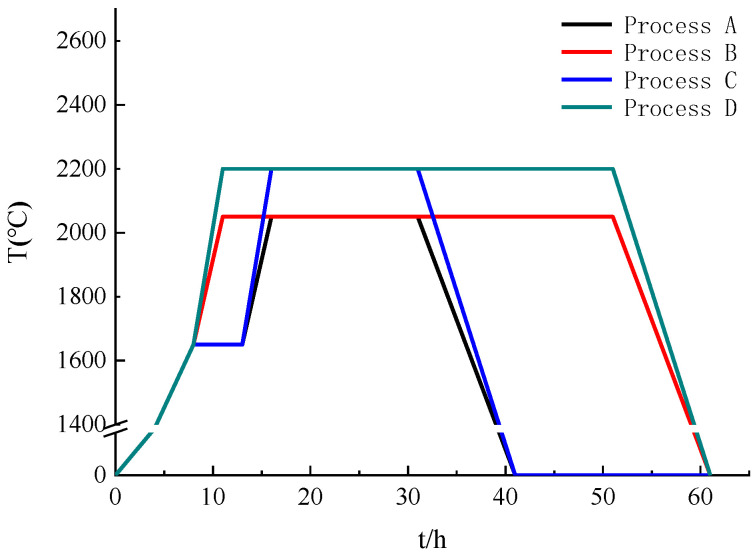
Curves of four processes.

During the powder heat treatment experiment, the raw materials were divided into four groups, A, B, C and D, with 500 g each, corresponding to four different processes. The process curves of the four groups are shown in Figure 3. Group A was first heated to 1300 °C and held for 5 h in a vacuum state. At the end of heat preservation in this stage, high-purity argon was introduced to maintain an air pressure of 100–150 torr, and then the temperature was raised to 1650 °C for 5 h. Then, the temperature was raised to 2050 °C for 15 h. In the final stage, after the heat preservation stage, the temperature was slowly lowered to room temperature (process A). Group B was directly heated to 2050 °C at a certain rate and held for 50 h. During this period, the furnace maintained a high-purity argon atmosphere with a pressure of 100–150 torr (process B). The process for the first half of group C was the same as that for group A until the heat preservation stage at 1650 °C was completed, and then the temperature was raised directly to 2200 °C, lasting for 15 h. During this period, high-purity argon was introduced into the furnace with a pressure of 100–150 torr (process C). Group D was heated directly to 2200 °C at a certain rate and held for 50 h. During this period, the furnace maintained a high-purity argon atmosphere with a pressure of 100–150 torr like in the other processes (process D).

**Figure 4:**
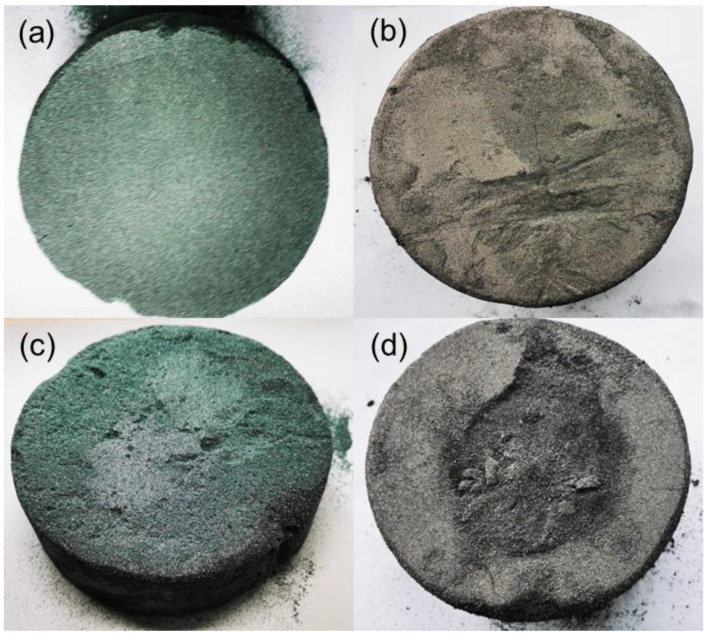
SiC powder after purification; (**a**) process A; (**b**) process B; (**c**) process C; (**d**) process D.

The state of raw materials after heat treatment by all four groups of purification processes is shown in Figure 4. Before processing, the SiC powder appeared as a green powder. After heat treatment in process A, the color of the powder turned dark green, and it was sintered from irregular particles into a massive solid, which was not very hard and could easily shed a few particles. In contrast, by prolonging the high-temperature period in process B, the powder color after heat treatment was significantly changed. The surface area became gray due to SiC graphitization, while the internal area was dark green with a hard block structure. In contrast, the powder was sintered into a ceramic state, and almost no SiC powder could fall off after process B. After heat treatment in process C, the color of the powder became dark green, which was darker than that in process A. The powder was sintered from irregular particles into a massive solid plate. There was a slight black graphitization phenomenon on the surface, and the powder was heavily ceramicized. The outer layer of the SiC powder after process D was seriously graphitized, while the porosity became larger. As the depth of the graphite layer increased, the outer layer of graphite became loose, which could be easily separated from the inner ceramic body. Compared with process B, the available volume of the ceramic parts in this scheme was smaller.

## 3. Results and Discussion

### 3.1. Graphitization and Polytype Change

**Figure 5:**
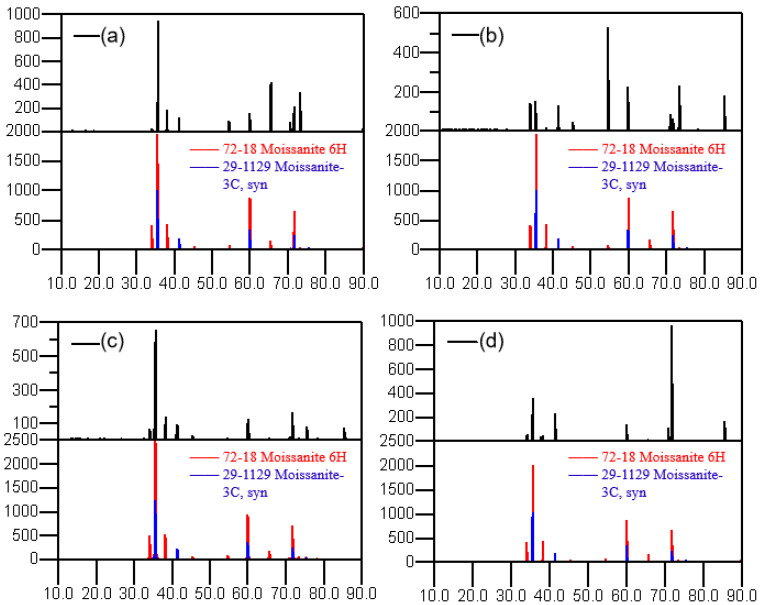
XRD patterns after different processes. (**a**) process A; (**b**) process B; (**c**) process C; (**d**) process D.

The data of the X-ray diffraction peaks were obtained using an X-ray diffractometer. The XRD patterns after heat treatment under different processes are shown in Figure 5. After process A, the spectral peak is not noticeably changed compared with the XRD spectral peak of the raw material, and the polytype is almost unchanged. From the state of the peak intensity, the proportions of 6H and 3C polytype crystals are relatively high, which indicates that the main crystal polytypes of the SiC powder after heat treatment in process A are still 6H and 3C. After process B, the intensity of the characteristic peak at 34.09° and 54.63° corresponding to the 6H polytype 2θ angle becomes stronger. Meanwhile, the peak intensity of 3C becomes weaker, indicating that its content has decreased, and the polytypes have changed to a certain extent under this condition. After process C, the SiC powder has both 6H and 3C polytypes. In contrast, the powder after process D has the obvious characteristic peak of a 6H polytype, while the characteristic peak of the 3C polytype is almost impossible to find. This indicates that after a 2200 °C high-temperature reaction for a long time in this process, the full recrystallization of raw materials occurs, and almost all 3C polytypes are converted to 6H polytypes under this condition.

### 3.2. Impurity Changes in the Powder

**Figure 6:**
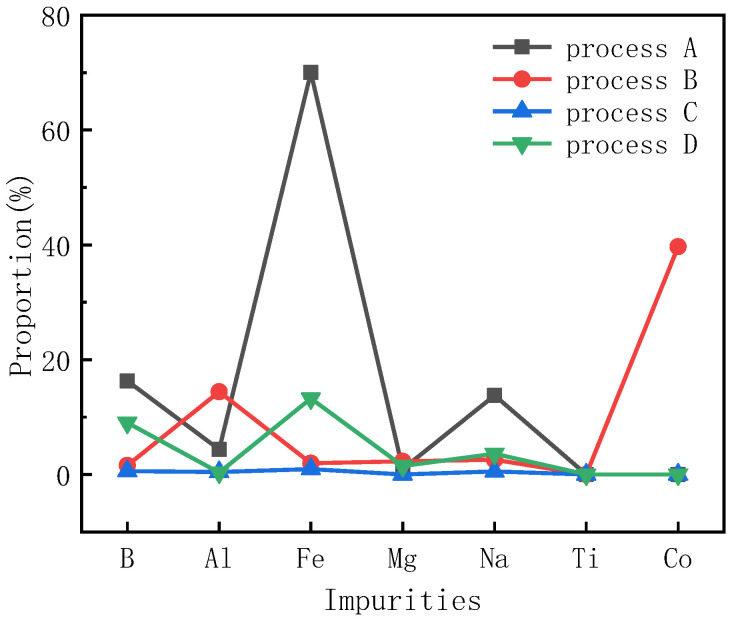
Proportion of sample impurities to impurities in raw powder.

A glow discharge mass spectrometer is a general piece of equipment used to detect the contents of impurities. Table 1 shows the GDMS data of the powders after heat treatment preparation by different purification processes. For comparison, the ratio of the impurity content in the sample after heat treatment to the impurity content in the initial sample was obtained, as shown in Figure 6. It should be pointed out that the concentrations of free Si, C and O in these samples were measured using special methods and equipment, such as heat treatment and NaOH corrosion, and were around 0.04%, 0.04% and 0.03%, respectively. After the heat treatment process, the contents of free Si and free O in the above components decreased to below 0.01%. Other impurity contents decreased to different extents.

After heat treatment in process A, the contents of the metal elements Al, Fe, Mg, Na, Ti and Co decreased significantly, and even the impurities of nonmetallic elements such as B also decreased significantly. Compared with process A, for the SiC powder after process B, the contents of metal elements Al, Fe, Mg, Na, Ti and Co were also greatly reduced. Among them, the content of the Fe impurity was further reduced, while the content of Al was only slightly reduced. The Al element was held at 1650 °C for 5 h in process A, and the SiC raw material needed to undergo high-temperature treatment in its normal preparation process. Impurities containing Al generally exist in the form of oxides and can react in large amounts at this temperature. Oxidized Al can decompose free Al_3_C_4_ formed by the C reaction decomposed from SiC and further form free Al under heating, which is sublimated into SiC powder. When the powder is directly heated to 2050 °C and has not experienced the 1650 °C step, Al impurities are likely to be doped in the lattice of SiC during the recrystallization of SiC grains, resulting in a lower impurity overflow than that in process A. The impurity of the nonmetallic element B also decreased significantly, and it decreased more than that in process A, which indicates that 1650 °C does not play a significant role in the volatilization of the B impurity, but the volatilization amount of element B needs to be greater in a longer high-temperature treatment.

Compared with processes A and B, the contents of metal elements Al, Fe, Mg, Na, Ti and Co in the SiC powder after heat treatment in process C were greatly reduced, in which the content of Fe impurities was further reduced to 1.15 ppm, and the content of Al was also reduced to 0.51 ppm. Process C is based on process A, and the second temperature step increases the temperature. In the early stages of the process, the powder is kept at 1650 °C for 5 h, and Al-containing impurities can react in large quantities at this temperature stage. In particular, Al_2_O_3_, Al(OH)_3_, Al_3_C_4_, etc., are common combined states of Al-containing impurities, which can be decomposed into Al_2_O_3_ and free Al at this temperature. Subsequently, at a later-stage temperature higher than that of process A, Al_2_O_3_ is further sublimated, and impurities containing Al are further reduced.

Compared with process C, the impurity contents of the metal element Fe in the SiC powder after heat treatment in process D also decreased, but the decrease was not as significant, which is consistent with the previous analyses. Compared with process B, although process D is at a higher temperature, except for Al and Fe impurities, the contents of other metal impurities decreased to a similar extent. At higher temperatures, since large quantities of SiC begin to decompose, Si and Fe mainly form liquid compounds or solid solutions at this temperature. After contacting decomposed graphite, they combine into compounds or solid solutions of C and Fe, which are also mostly liquid at this temperature. At the same time, during the decomposition and recrystallization of SiC, the element F continuously combines with Si and C in SiC, which also causes some Fe to be retained in the ceramic body of recrystallized SiC. This is why the decrease in Fe content is smaller than that of process B. In process B, at 2050 °C, the decomposition rate of SiC is slower, and the recrystallization process is much weaker than that of process D. The liquid phase of Fe and Si or Fe and C at low pressure is less involved in recrystallization than that of process D, and more of the material is sublimated when the powder pores are larger. At a higher temperature, due to the decomposition of SiC, more Si is ionized with Al, and the vaporization point of this component is low, which leaves powder in the gas phase. The nonmetallic element B at a direct high temperature is similar to the rule of the Fe element, and it is more difficult to sublimate and discharge. The compounds B and C are usually stable, and the vaporization point is high. Therefore, the effect of impurity removal in process D is not as good as that in process B. After sublimation, Si combines with B to synthesize SiB_3_ and other compounds, which sublimate and volatilize at low pressure. However, during this later stage, these compounds will be trapped inside the raw material due to the ceramicization of SiC. After process C, the element B and the lower-melting-point metal are melted together to form alloys and compounds, and then the powder sublimates from the framework of porous SiC once the temperature is raised, which helps to remove impurities.

Table 2 shows the mass loss of raw materials after the heat treatment processes. The mass loss of group D reached 50% due to a large amount of powder decomposition and graphitization, which greatly reduces the availability of raw powder materials. The material loss of group B reached nearly 30%, which indicates that the powder loss is serious under the condition of a single temperature step for a long time, and more solid SiC decomposes into gas phase components. Although the mass loss of group A is the smallest, its impurity removal effect is also insufficient and cannot meet the requirements of high-purity raw materials. In contrast, the loss of raw materials in the group C powder process is relatively small, which is 6.7%. Considering the results of the GDMS test in Table 2, the purification effect of process C is excellent, and the purified SiC materials are more suitable for the high-purity raw materials required for the growth of high-quality silicon carbide crystals.

## 4. Conclusions

Commercial SiC powder contains high concentrations of free Si, free C, free O, Al, B and small amounts of metal impurities. Among them, some impurities will be deposited into the crystal during the subsequent growth of single-crystal SiC, forming voids caused by Si-containing impurities, and carbon inclusions will form a large number of defects and dislocations. Therefore, commercial SiC powder can be used as the raw material for subsequent single-crystal growth only after high-temperature heat treatment and purification. In this study, powder heat treatment processes with different time lengths and different temperature steps were carried out, and the sintered samples were analyzed using XRD and GDMS methods. The results show that under the designed high-temperature and long-term processing conditions, the SiC powder will undergo obvious sublimation and recrystallization during the heat treatment and purification process. The polytype is mainly transformed into 6H. The impurities in the powder source are substantially volatilized, and the purification effect is good, but the powder loss is serious. The mass loss of purified powder by low-temperature and short-time heat treatment is almost negligible, only removing the impurities on the surface of the powder source, and the effect of removing impurities is poor. Finally, heat treatment process A ingeniously combines the advantages of different temperature steps with a good purification effect, and less material is consumed. As a result, a high-purity SiC source with nearly 5N purity was successfully prepared with a mass loss of 6.7%, free Si, free C and free O all less than 0.01% and the contents of impurities such as Al, B, Fe, Mg, Na and Ti less than 1.15 ppm, which is close to the ppb levels observed in the present study.

## Figures and Tables

**Table 1:** GDMS data of different powders.

Impurities	Original Powder	Powder after Process A	Powder after Process B	Powder after Process C	Powder after Process D
B	30 ppm	4.89 ppm	0.48 ppm	0.18 ppm	2.71 ppm
Al	114 ppm	4.98 ppm	16.45 ppm	0.51 ppm	0.38 ppm
Fe	120 ppm	84.01 ppm	2.36 ppm	1.15 ppm	15.91 ppm
Mg	50 ppm	0.47 ppm	1.15 ppm	0.005 ppm	0.74 ppm
Na	40 ppm	5.51 ppm	1.03 ppm	0.22 ppm	1.45 ppm
Ti	30 ppm	<0.005 ppm	<0.005 ppm	<0.005 ppm	<0.005 ppm
Co	25 ppm	<0.005 ppm	9.92 ppm	<0.005 ppm	<0.005 ppm

**Table 2:** Quality change of raw materials before and after heat treatment.

Process	Before Process/g	After Process/g	Mass Loss/%
A	500	492.6	1.5%
B	500	351.4	29.7%
C	500	466.5	6.7%
D	500	232.9	53.4%

## Data Availability

Data is contained within the article.
